# Nitrite of Amyl

**Published:** 1892-06

**Authors:** Henry A. Kelley


### NITRITE OF AMYL.[1]

BY HENRY A. KELLEY, D.M.D.

This drug has received considerable attention from the medical profession during the past few years, and is of interest to dentists, as it is one of the best antagonists to cocaine, and is so recommended by many of our authorities. It is also used very successfully in

---

[1] Read before the Harvard Odontological Society, December 23, 1891.

cases of collapse from anæsthetics, and in hysteria. I might enumerate a great many uses for the drug, but suffice to say, it is a powerful antispasmodic, and whenever indicated, nitrite of amyl will be found useful.

The formula of amyl nitris is,—

$$C_5H_{11}NO_2$$

| | |
|---|---|
| Sp. gr. . . . | 0.877 |
| Boiling point . . | 182° F. |

It is obtained by heating one part of strong $HNO_3$ with two parts of rectified fusel oil, until reaction commences, when the heat is withdrawn and afterwards reapplied. The distilled portion, obtained below 212° F. is rectified by means of carbonate of potassium, and that portion only distilled between 202° and 206° F. is reserved, being a nitrite of the oxide of amyl.

Nitrite of amyl is a yellow liquid, very volatile, and with an odor which some liken to over-ripe pears, but which I always remember by its similarity to that of the squash-bug. If you have ever been near that animal, you know the odor; and I feel sure, when once you have become familiar with it, you will never mistake it.

The best test for it is its most striking property, that of flushing the face when inhaled. There is also a sense of fulness, of giddiness, and sometimes syncope, which latter is due to the sudden rush of blood to the periphery, thus drawing it from the brain. This flushing of the face was first noticed by Dr. Guthrie, in 1859. It was not until 1866 that attention was again called to the drug by Dr. Richardson. Dr. Richardson presented the following conclusions:

"1. It is absorbed by the skin, stomach, lungs, and cellular tissue.

"2. After absorption its effects are seen immediately on the heart and circulation, producing, first, violent action of the heart and dilatation of the capillaries; followed, secondly, by diminished power of the heart and contraction of the extreme vessels.

"3. In man it can reduce the forces of respiration and circulation to the extent of a trance or catalepsy.

"4. It is not an anæsthetic; by it consciousness is never destroyed, unless a condition approaching death be produced.

"5. The effects of the nitrite are through the motor force, which it wildly excites and then subdues.

"6. The *modus operandi* of the drug appears to be by arresting the process of oxidation of the tissue.

"7. Physically the nitrite is between the volatile and solid bodies. Hence its effects are less effervescent than those of very volatile substances, as ether, chloroform, etc., and less certainly destructive than the solid substances, as opium, etc. In this lies the secret of its prolonged action."

It also paralyzes the nerves from periphery to centre, lessening the contractile power of the arteries.

Dr. Wood disputes the fact of its producing a great excitation of the motor system; he having found from his experiments that it produces a gradually-increasing paralysis. In regard to Dr. Richardson's other experiments, we will leave them until we come to those of other men, some of whom will confirm and others dispute them.

In regard to the trance, which we will find disputed, it is but fair to say Dr. Richardson observed animals lie in this state for many hours. You will also please remember what he says about its not being an anæsthetic, and its acting by arrest of oxidation of the tissues.

Dr. Brunton has, perhaps, investigated this subject as thoroughly as any experimenter up to the present time. They were performed in the laboratory of and observed by Professor Luding, of Leipsic, and must carry great weight. They are mostly upon rabbits. The animals were not allowed to breathe the vapor directly, but were supplied, artificially, by an apparatus so arranged that air might be sent directly through the bellows and tube into the lungs, or passed through a vessel containing the vapor of nitrite of amyl.

The bellows being worked by an engine with great regularity, an even respiration was produced, and the disturbing influences on the blood were in a great measure avoided.

Uneven respiration causes suspension of this function for a short time when any strong-smelling vapor, such as we know nitrite of amyl to be, is administered. Also by the alteration in the condition of the blood, caused by this uneven respiration, irritation of the vagus, and thus slowing of the heart is produced.

The vagus being the inhibitory nerve of the heart, any irritation or stimulation must cause slowing of the heart-beat.

On the inhalation of air charged with the nitrite the blood-pressure rapidly sank, the pulse-rate being unaffected.

On the continuance of the administration the pressure rose

After twenty seconds the vapor was discontinued, and the pressure rose even more strongly, and at the longest attained its normal height in one minute.

This experiment demonstrated that small quantities produce great effects, soon passing off; becoming either excreted or destroyed in the body.

To counteract the effects of convulsions experienced by the animal, and which it was thought affected the results, the next one was treated with curare; a poison whose action is to paralyze the voluntary but not the involuntary muscles.

Under these conditions the pressure sank at once, as in the previous experiment; but, unlike it, did not return to the normal as long as the animal was given the drug. These convulsions have very seldom been noticed in man under the influence of amyl. Now, this lowering of the blood-pressure, is it caused by the lessened power of the heart, or by the dilatation of the arteries? It is due to the dilatation of the arteries, for the same flushing may be produced by the section of the sympathetic. This section paralyzes the vaso-constrictors, which give the "tone," and thus allows dilatation of the arteries. This is well illustrated, experimentally, by section of the sympathetic in a rabbit, and noting the flushing of the ear. This tone of the arteries I have spoken of is probably due to a certain constriction of the muscular, or middle, coat, but this is regulated and augmented by the so-called vaso-motors; and here, if you will permit me, I would like to refresh your memory in regard to the vaso-motor nerves. There is one class of these nerves called vaso-dilators, the irritation of which always causes dilatation, as the chorda tympani; another class called vaso-constrictors, for they always constrict the arteries when irritated, as the cervical sympathetic; and a third class, sometimes causing dilatation, and at other times constriction, as the sciatic. Perhaps we should not call this last a class by itself, for it is due simply to the fact that some nerves, such as the sciatic, carry fibres of both vaso-dilators and vaso-constrictors.

Now, it must be plain, if, as in the experiment, we cut the sympathetic, which has vaso-constrictor fibres in it, we paralyze the vaso-constrictors which give the tone to the arteries of the rabbit's ear, for this is the nerve that is distributed to those arteries. When this constriction is removed these must dilate, and we see the flushing, which, as I said, must be due to their dilatation. Having decided this point, the discussion must proceed to another not so easily disposed of.

Is this dilatation caused by the direct action of the amyl on the walls of the arteries, or through the vaso-motor centre in the medulla oblongata?

The almost universal belief was, at first, in favor of the action through the vaso-motors, and this view was taken by Drs. Richardson and Waldo. This theory has to-day many supporters. Brunton admits they may play a small part, but does not believe in them as the principal cause.

Dr. Waldo's reasons for supporting the vaso-motor theory were that the flushing which follows the use of amyl is nearly absent in paralytics, in whom the whole motor system is paralyzed; while in epileptics, in whom the vaso-motor nerves are very sensitive, the flush is very decided.

But, from my study, I am led to the opinion that it is through the walls of the arteries the nitrite acts. Let us look at Brunton's experiment on this point.

Now, provided the nitrite acts through the vaso-motor centres in the brain, the nitrite could not possibly have any effect if these centres be separated from the vessels by cutting the cord at the neck. But if, on the other hand, it acts directly on the vessels, this cutting should not affect the result of the administration of the vapor. So, from this reasoning, Brunton cut the cord, and immediately the blood-tension sank very low, owing to the loss of tone of the arteries, they being cut off from their vaso-motors. He then gave the amyl, and observed a still further sinking of the tension. And this lowering of the tension bore the same relation to the existing tension before giving the vapor that it did in experiments performed without cutting the vaso-motors.

This seems to me a very conclusive experiment in favor of its action on the walls of the arteries. The only objection I can see to the experiment is the fact that he does not cut off all his vaso-motor centres—for there are such centres—down the spinal cord. But these are not usually active, and so may practically be excluded.

I will now call your attention to a few experiments made by Dr. H. C. Wood. These experiments I take from his essay, successfully competing for a prize offered by the Massachusetts General Hospital for original work.

A kitten was given twenty to thirty drops by inhalation. It died in a few moments without struggling. At immediate autopsy the heart was found beating strong, and continued to beat vigorously for ten minutes. Blood was found of a dark color; no distinction between venous and arterial blood being made out. In all his experiments this dark blood was present when the experiment was carried to death. Here is a severe blow to that favorite theory of some to account for anæsthesia,—namely, by withholding oxygen from the blood. Here is a drug which withholds the oxygen to such an extent that the venous and arterial blood cannot be distinguished, and yet is not an anæsthetic.

Dr. Wood did not observe the trance-like condition observed by Dr. Richardson, and he claims consciousness is always lost very late, and not early.

In all his experiments there was a great loss of muscular power. Now, this may have happened in four ways:

1. Impairment of the will-power to arouse the motor-ganglia of the spine.
2. Impairment of the power of said ganglia.
3. Impairment of the conducting power of the nerves.
4. Impairment of the contractile power of the muscles.

In answer to the first and second, I may say there is no proof of the drug possessing such peculiar powers. So we will dismiss them at once as to the action on the conducting power of the nerves and the contractility of the muscles. Wood demonstrated in fatal doses that it affects but does not destroy the conductility of the nerves. He then showed that the local application of the amyl destroyed the contractile power of the muscles. Therefore, the nitrite acted to paralyze the muscles, and this accounts for the great loss of power manifested.

This has an important bearing on the question, whether the vaso-motor nerves do or do not cause the dilatation of the arteries.

Dr. Wood agrees with Brunton that the nitrite of amyl diminishes the blood-pressure; that the division of the vagus does not affect this result, and that it still acts after the division of the spinal cord at the neck.

Now, of the four ways in which the circulation of the blood can be affected by this drug:

(I recognize, of course, that there are many more ways in which the circulation of the blood is influenced, but I do not think they are sufficient to produce the great lowering of tension we see produced by the action of the amyl.)

1. It may be influenced by the vagus.
2. By the accelerator nerves.

Both of these, I have shown, are not the cause.

3. By acting on the muscles of the heart. That this is not the true cause in this case may be shown by compressing the aorta in the abdomen, after dividing the spinal cord in the neck. Thus the flow of blood to the lower extremities is checked, but on inhalation a rising, though more generally a sinking, pressure (which is much less than in the normal) occurs.

If the nitrite acted on the heart, the compressing of the arteries would not affect the result. Therefore, it must act on the capillaries.

4. This it does, in my belief, by paralyzing the muscular coat of the arteries, as I called your attention to its being able to paralyze muscle when applied locally.

If it can cause this complete paralysis in fatal doses, it is fair, I think, to suppose it can cause a partial paralysis in moderate doses, and thus produce a dilatation; hence the low tension observed on the blood-circulation.

It is usually administered by inhalation, and is comparatively harmless.

Weak and nervous people are very susceptible to its influence, and care should be taken when using it with them. A small dose should first be given as an indicator. Like many other drugs, a patient may become accustomed to its presence, and be able to tolerate large doses after inhaling it regularly. Persons using it for angina pectoris find, in order to get the relief they look for, that they are obliged to increase the dose, and, finally, there are some cases where it has ceased to benefit.

This use of nitrite of amyl in angina pectoris is an illustration of a theory which worked well in practice. Brunton knew the distinctive property of this drug was to lower the blood-tension, and also that the disease, angina pectoris, was always accompanied by a high blood-tension. Therefore he prescribed nitrite of amyl, with what results can be told you by the many victims of the disease who break their capsule of amyl nitrite on their handkerchief when warned of its appearance, and thus prevent the threatened attack.

It is usually administered by inhalation, and as it deteriorates on exposure to the air, it is best kept in little glass capsules or pearls, three or five drops. These are broken on the handkerchief and then applied to the nostril.

The dose usually given by inhalation is from two to three drops. Not more than three should be given, unless the patient is accustomed to its use. I consider five as large a dose as it is advisable to give, and usually find two sufficient. It is administered by the stomach in doses from one-quarter to one minim; but the results are much more satisfactory when inhaled.

The antagonists to this drug are ergot, belladonna, and other spinal stimulants.

